# Transcriptomic Profile Analysis of Brain Tissue in the Absence of Functional TRPM8 Calcium Channel

**DOI:** 10.3390/biomedicines13010075

**Published:** 2024-12-31

**Authors:** Erick B. Saldes, Alexandra Erdmier, Jai Velpula, Timothy E. Koeltzow, Michael X. Zhu, Swapna Asuthkar

**Affiliations:** 1 Department of Cancer Biology and Pharmacology, University of Illinois College of Medicine Peoria, Peoria, IL 61605, USA; esaldes@uic.edu (E.B.S.); aerdmier@uic.edu (A.E.); tkoeltzow@fsmail.bradley.edu (T.E.K.); 2 Pringle Robotics, Peoria, IL 61605, USA; jaivelpula@gmail.com; 3 Department of Psychology, Bradley University, Peoria, IL 61625, USA; 4 Department of Integrative Biology and Pharmacology, McGovern Medical School, The University of Texas Health Science Center at Houston, Houston, TX 77030, USA; michael.x.zhu@uth.tmc.edu; 5 Department of Pediatrics, University of Illinois College of Medicine Peoria, Peoria, IL 61605, USA

**Keywords:** TRPM8, mRNA, LncRNA, methylation, androgen, brain, behavior

## Abstract

**Background/Objectives:** Transient Receptor Potential Melastatin 8 (TRPM8) is a non-selective, Ca^2+^-permeable cation channel involved in thermoregulation and other physiological processes, such as basal tear secretion, cell differentiation, and insulin homeostasis. The activation and deactivation of TRPM8 occur through genetic modifications, channel interactions, and signaling cascades. Recent evidence suggests a significant role of TRPM8 in the hypothalamus and amygdala related to pain sensation and sexual behavior. Notably, TRPM8 has been implicated in neuropathic pain, migraines, and neurodegenerative diseases such as Parkinson’s disease. Our laboratory has identified testosterone as a high-affinity ligand of TRPM8. TRPM8 deficiency appears to influence behavioral traits in mice, like increased aggression and deficits in sexual satiety. Here, we aim to explore the pathways altered in brain tissues of TRPM8-deficient mice using the expression and methylation profiles of messenger RNA (mRNA) and long non-coding RNA (lncRNA). Specifically, we focused on brain regions integral to behavioral and hormonal control, including the olfactory bulb, hypothalamus, amygdala, and insula. **Methods:** RNA was isolated and purified for microarray analysis collected from male wild-type and TRPM8 knockout mice. **Results:** We identified various differentially expressed genes tied to multiple signaling pathways. Among them, the androgen–estrogen receptor (AR-ER) pathway, steroidogenesis pathway, sexual reward pathway, and cocaine reward pathway are particularly worth noting. **Conclusions:** These results should bridge the existing gaps in the knowledge regarding TRPM8 and inform potential targets for future studies to elucidate its role in the behavior changes and pathology of the diseases associated with TRPM8 activity.

## 1. Introduction

The Transient Receptor Potential Melastatin 8 (TRPM8) protein not only plays a well-established role in thermoregulation but also has been implicated in a broad range of physiological functions, including basal tear secretion, cell differentiation, and insulin homeostasis [1,2,3]. TRPM8 protein has been identified in the somata of a variety of central nervous system (CNS) structures, including cortical, limbic, basal ganglia, thalamic, and midbrain structures [3] Thus, it is not surprising that TRPM8 is implicated in a range of neurological and behavioral conditions, including migraine, neuropathic pain, Parkinson’s disease, and epilepsy [4,5,6]. Indeed, a prior bioinformatics report highlighted the role of TRPM8 as a potential biomarker for migraine susceptibility [7].

We have previously identified TRPM8 as a high-affinity target of testosterone, representing a novel mechanism by which this sex hormone can rapidly influence neural activity [8,9,10]. Notably, mice lacking the *Trpm8* gene exhibit significant disruptions in sexual behavior, as well as aggression and elevated testosterone levels after mating [10]. In addition, female *Trpm8* null mutant mice exhibit perseverative sniffing behavior to male pheromones, while the male mutant mice display disrupted midbrain dopamine activity and fail to respond to testosterone, with elevated neuronal activity in the amygdala typically seen in male wild-type mice [10]. Taken together, these data highlight the possibility that TRPM8 is pivotal to sexual behavior as it is well established that the amygdala, along with the olfactory bulb and the hypothalamus, plays central roles in rodent sexual behavior [11,12,13]. Also, the insular cortex is similarly implicated in sex differences of several CNS conditions, modulated by midbrain dopamine, and commonly inclusive of the substantia innominata in brain dissection, an area reported to express high levels of *Trpm8* mRNA [14,15,16,17,18,19].

Disruption of TRPM8 activity in mice may precipitate a range of physiological and behavioral changes by influencing the transcriptomic landscape of brain areas essential to motivated behavior. In contrast to TRPM8, which has been well characterized in the periphery and in association with sensory processing [20], there is a paucity of research examining the role of TRPM8 in motivated behavior. To better understand the impact of the TRPM8 deletion on sexual behavior, the present study aims to characterize the gene expression profile changes of *Trpm8* mutant mice using microarray. This analysis provides insights into the alterations in signaling pathways related to long non-coding RNAs (lncRNAs) and messenger RNAs (mRNAs). We included the olfactory bulb, amygdala, hypothalamus, and insula in the analysis due to their known involvement in rodent sexual behavior and reward. Our findings reveal a multitude of differentially expressed genes that modulate a variety of neural signaling pathways associated with various disorders. This RNA-Seq dataset establishes a foundation for future investigations exploring the interactions of TRPM8 and genes identified here, which may further unveil sex differences across a variety of disorders.

## 2. Materials and Methods

### 2.1. Ethics Statement

The Institutional Animal Care and Use Committee of the University of Illinois College of Medicine at Peoria, Peoria, IL, USA, approved all surgical interventions and post-operative animal care. The consent was written and approved. The accepted protocol number is 1456039, dated 18 May 2022 and renewed on 18 June 2024.

### 2.2. Animals and Housing Conditions

Male C57BL/6J (WT, #000664) and TRPM8 knockout (KO, #008198) mice were obtained from Jackson Laboratories (Bar Harbor, ME) and were directly utilized for RNA microarray analysis. The TRPM8 KO mice were originally donated to Jackson Laboratories by Dr. David Julius’ laboratory [21]. These mice undergo regular genotype evaluations in accordance with the Jackson Laboratory genotyping protocol. In our study, we maintained the mice without any breeding. Mice were housed in groups of 2–7 per cage in yellow-tinted plastic cages (43.2 cm length × 20.3 cm width × 20.3 cm height) containing structural enrichment (nesting material and a cardboard tube/hiding place) at the University of Illinois College of Medicine in Peoria. Mice were kept under a 12/12 light/dark cycle (lights on at 06:00 and off at 18:00 h) under consistent temperature and humidity (22.8 ± 2.0 °C, 45–50% humidity) with food and water available ad libitum (LabDiet 5LG4). At 14 weeks old, mice were euthanized by CO_2_ asphyxiation and cervical dislocation. The brain tissue was harvested and snap-frozen. The specific brain regions were dissected and processed for RNA isolation and microarray analysis.

### 2.3. RNA Isolation and Purification

RNA was isolated from brain tissues of WT and TRPM8 KO mice using the TRIzol reagent (Invitrogen, Carlsbad, CA, USA). This method is widely recognized for its efficiency in extracting high-quality RNA from various tissues, including those with complex compositions [22]. The brain regions analyzed included the hypothalamus, olfactory bulb, amygdala, and insula, selected based on their involvement in physiological and behavioral responses previously reported to be affected in TRPM8 KO mice. Tissue samples from each mouse were pulled and homogenized in TRIzol reagent following the manufacturer’s protocol. The homogenate was incubated at room temperature for 5 min to allow for complete lysis of the cells. Subsequently, chloroform was added to the mixture, and the samples were shaken vigorously for 15 s before being allowed to stand for 2–3 min. The samples were then centrifuged at 12,000× *g* for 15 min at 4 °C. The aqueous phase containing the RNA was carefully transferred to a new tube, and RNA was precipitated by adding isopropanol and incubating at −20 °C for at least 1 h. Following centrifugation, the RNA pellet was washed with 75% ethanol, air-dried, and resuspended in RNase-free water. The quality and quantity of the isolated RNA were assessed using a NanoDrop ND-1000 (ThermoScientific, Waltham, MA, USA). The purity of RNA was evaluated by measuring the absorbance ratios at 260/280 nm and 260/230 nm, with ideal ratios being approximately 2.0 [23]. RNA integrity was confirmed through agarose gel electrophoresis. Following RNA isolation, the samples were sent to ArrayStar (Rockville, MD, USA) for microarray-based mRNA/LncRNA and epi mRNA/LncRNA methylation analysis. This comprehensive analysis allows for the simultaneous assessment of gene expression and epigenetic modifications, providing insights into the regulatory mechanisms underlying differential gene expression in the context of TRPM8 KO.

### 2.4. RNA and LncRNA Labeling and Array Hybridization

Sample labeling and array hybridization were performed according to the Agilent One-Color Microarray-Based Gene Expression Analysis protocol (Agilent Technology, Santa Clara, CA, USA), with minor modifications. Briefly, each RNA sample was amplified and transcribed into fluorescent cRNA along the entire length of the transcripts without 3′ bias utilizing a mixture of oligo(dT) and random priming method (Arraystar Flash RNA Labeling Kit, Arraystar, Rockville, MD, USA). The labeled cRNAs were purified using an RNeasy Mini Kit (Qiagen, Hilden, Germany). The concentration and specific activity of the labeled cRNAs (pmol Cy3/μg cRNA) were measured with NanoDrop ND-1000 (ThermoScientific, Waltham, MA, USA). Briefly, 1 μg of each labeled cRNA was fragmented by adding 5 μL 10 × blocking agent and 1 μL of 25 × fragmentation buffer; then, the mixture was heated at 60 °C for 30 min, and finally, 25 μL 2 × GE hybridization buffer was added to dilute the labeled cRNA. A hybridization solution (50 μL) was dispensed into the gasket slide and assembled to the LncRNA expression microarray slide. The slides were incubated for 17 h at 65 °C in an Agilent Hybridization Oven. The hybridized arrays were washed, fixed, and scanned by using the Agilent DNA Microarray Scanner (part number G2505C).

### 2.5. RNA and LncRNA Data Analysis

Agilent Feature Extraction software (version 11.0.1.1) was used to analyze acquired array images. Quantile normalization and subsequent data processing were performed with the GeneSpring GX v12.1 software package (Agilent Technology, Santa Clara, CA, USA). After the quantile normalization of the raw data, LncRNAs and mRNAs with at least 3 out of 6 samples having flags in Present or Marginal (“All Targets Value”) were chosen for further data analysis. Differentially expressed LncRNAs and mRNAs with statistical significance were identified through Volcano Plot filtering between two groups. Hierarchical clustering was performed on log2-transformed normalized array intensity values scaled for z-score across the samples using heatmap.2 R software package (gplots version 2.3.2) [24]. GO analysis and Pathway analysis were performed using the topGO Bioconductor package. Expression data were sent to PharmaFace (Begumpet, Hyderabad, India). The correlative analysis was conducted using scatter plots that depict the expression changes of mRNAs and lncRNAs, utilizing log2 fold change values. The categorization of genes was based on their expression trends, allowing us to identify relationships between the two RNA classes. The scatter plot was generated using R version 4.4.1 (R Foundation for Statistical Computing, Vienna, Austria) using ggplot2 v3.5.1 [24].

### 2.6. Methylation RNA Labeling and Hybridization

The anti-m6A antibody immunoprecipitated “IP” RNAs and the corresponding supernatant “Sup” RNAs were added with an equal amount of calibration spike-in control RNA and then separately amplified and labeled with Cy3 (for “Sup”) and Cy5 (for “IP”) using Arraystar Super RNA Labeling Kit. The synthesized cRNAs were purified using an RNeasy Mini Kit. The concentration and specific activity (pmol dye/μg cRNA) were measured with NanoDrop ND-1000. Cy3 and Cy5 labeled cRNAs (2.5 μg) were mixed. The cRNA mixture was fragmented by adding 5 μL 10° blocking agent and 1 μL of 25° fragmentation buffer, heated at 60 °C for 30 min, and combined with 25 μL 2° hybridization buffer. A hybridization solution (50 μL) was dispensed into the gasket slide and assembled to the m6A-mRNA&lncRNA Epitranscriptomic Microarray slide. The slides were incubated at 65 °C for 17 h in an Agilent Hybridization Oven. The hybridized arrays were washed, fixed, and scanned using an Agilent Scanner G2505C.

### 2.7. Methylation RNA Data Analysis

Agilent Feature Extraction software (version 11.0.1.1) was used to analyze the acquired array images. Raw intensities of IP (immunoprecipitated, Cy5-labeled) and Sup (supernatant, Cy3-labelled) were normalized with an average of log2-scaled spike-in RNA intensities. After spike-in normalization, the probe signals having present or marginal QC flags in at least 3 out of 6 samples were retained for further “m6A quantity” analyses. “m6A quantity” was calculated for the m6A methylation amount based on the IP (Cy5-labeled)-normalized intensities. Differentially m6A–methylated RNAs between two comparison groups were identified by filtering with the fold change and statistical significance (*p*-value) thresholds. Hierarchical clustering was performed on log2-transformed normalized m6A quantity values scaled for z-score across the samples using heatmap.2 R software (gplots version 2.3.2) [24]. Gene Ontology (GO) and KEGG pathway analysis were performed using the topGO R package (version 2.24.0), where the *p*-values were calculated by Fisher’s exact test.

### 2.8. Statistical Analysis

All data are from three independent samples. Student’s *t*-tests were used for comparisons. Statistical differences are presented at ns, *p* < 0.05, *, *p* < 0.05. Statistical analyses were performed using Minitab software Version 21.1.0 (Minitab, State College, PA, USA). 

## 3. Results

### 3.1. Identification of Differentially Expressed lncRNA and mRNA

**Figure 1:**
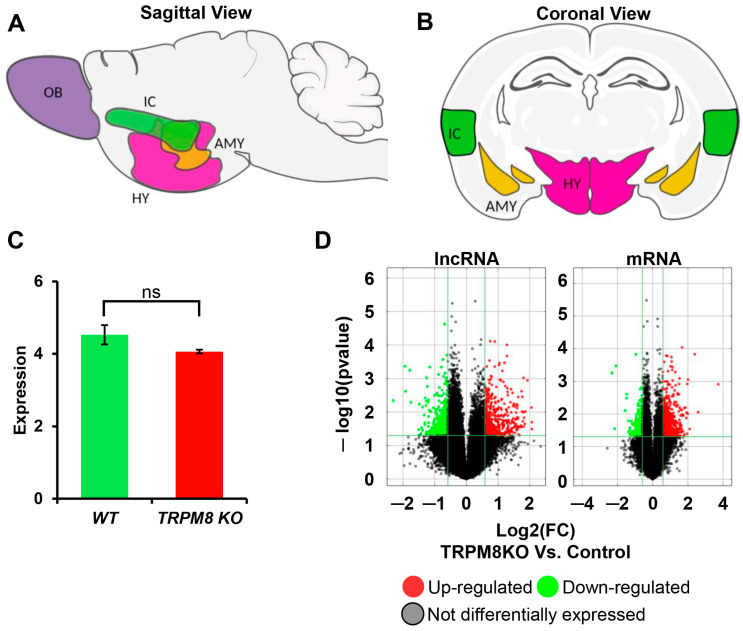
Differential expression analysis of lncRNA and mRNA of TRPM8 KO 14-week-old mice brain tissue: (**A**,**B**). Visual representation in a sagittal and coronal view of brain regions used from WT and TRPM8 KO for RNA sequencing analysis: olfactory bulb (OB; purple), amygdala (AMY; yellow), hypothalamus (HY; pink) and insular cortex (IC; green). (**C**). Microarray data showing the expression of mRNA between WT and TRPM8 KO (Student’s *t*-test: ns, *p* > 0.05; *n* = 3). (**D**). Volcano plots of DE lncRNAs and mRNAs (upregulated; red, downregulated; yellow, and not differentially expressed; gray/black).

RNA sequencing analysis to identify differentially expressed genes was performed by pulling together the hypothalamus, olfactory bulb, amygdala, and insula (Figure 1A,B) of WT and TRPM8 KO mice. These structures of the mouse brains were selected because of their involvement in a wide range of physiological and behavioral responses previously observed to be affected in TRPM8 KO mice [10]. Specifically, the hypothalamus regulates hormone production, the olfactory bulb is essential for smell, the amygdala controls emotional responses, and the insula integrates bodily sensations with emotional states [25,26,27,28]. Microarray analysis showed a slight reduction in mRNA expression in the TRPM8 KO compared to the WT (Figure 1C: Student’s *t*-test: ns, *p* > 0.05; *n* = 3). While TRPM8 mRNA is detectable in the TRPM8 KO, the TRPM8 protein is nonfunctional, as corroborated by findings from Dr. David Julius’ laboratory and Jackson Laboratories [21,29,30,31,32,33,34,35].

Our analysis primarily investigated the differentially expressed lncRNA and mRNA in TRPM8 KO versus WT brain tissues. We used volcano plot analysis to reveal differentially expressed genes (DEGs) in lncRNA and mRNA between the TRPM8 KO and WT control samples. The results showed that for lncRNA, 461 were upregulated, 546 were downregulated, and 29,492 were not differentially expressed. For mRNA, 661 were upregulated, 254 were downregulated, and 18,915 were not DEGs (Figure 1D). The DEGs between lncRNAs and mRNAs suggest a functional interplay, where lncRNA may influence the expression or function of their associated mRNAs. This implicates a cooperative role in regulating gene expression and cellular activities. It is possible that the TRPM8 channel affects signaling pathways that regulate the expression levels of specific lncRNA and their adjacent or nearby mRNA-coding genes [36]. Alternatively, lncRNAs may recruit chromatin-modifying complexes to specific genomic locations, which can either enhance or repress the transcription of mRNA-coding genes at such locations through changes in chromatin structure and accessibility [37,38]. Due to the role of TRPM8 as a channel/receptor of testosterone, it is also possible that lncRNAs and mRNAs show a coordinated expression under specific conditions to help compensate for changes in testosterone levels. Since lncRNAs can regulate the stability of mRNA via interactions with RNA-binding proteins, the loss of TRPM8 expression/function could amplify the associated transcription changes between certain lncRNAs and mRNAs [39].

**Figure 2:**
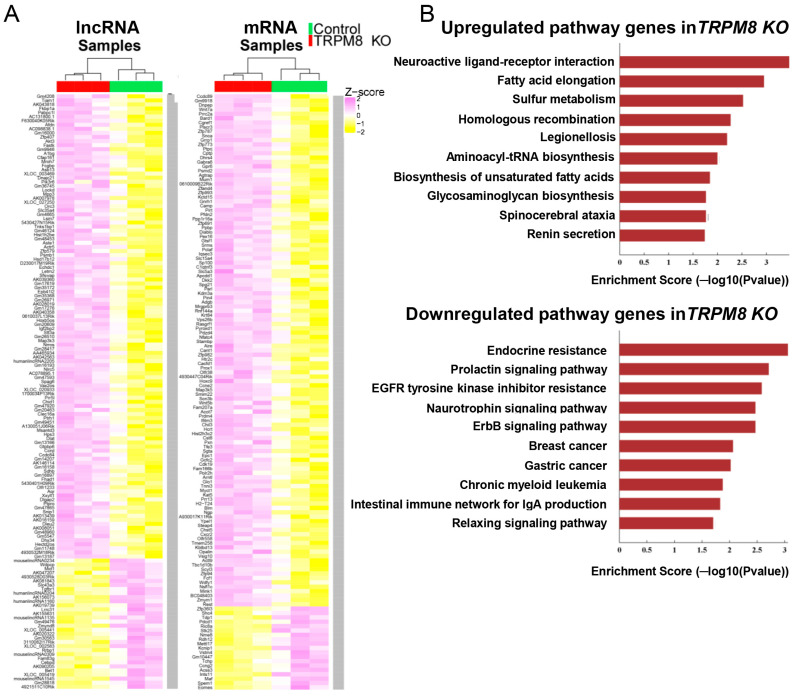
Heatmap visualization and pathway analysis of genes in TRPM8 KO brain tissue: (**A**). Heatmaps indicate the z-score of the differentially expressed genes from lncRNA and mRNA between control and TRPM8-KO. (**B**). Pathway analysis by mapping genes to the KEGG pathways shows the upregulated and downregulated signaling pathways in TRPM8 KO mice brains compared to WT control.

Figure 2A and Supplementary Table S1 shows selected DEGs in lncRNAs and mRNAs between TRPM8 KO and WT control samples ranked based on *p*-values with a cut-off of 0.05. The heatmaps were generated using the ComplexHeatmap library to better visualize clustering and differential expression. The mRNA and lncRNA were then pooled together before a pathway analysis was conducted mapping the DEGs to KEGG pathways. The significance of the pathway was determined by the *p*-value using Fisher’s exact test, where the lower the *p*-value, the more significant the pathway; a cut-off of 0.05 was used for both the upregulated and downregulated genes. Based on the enrichment score, the top ten pathways were graphed and are shown for both the upregulated and downregulated genes in the TRPM8 KO samples (Figure 2B). The complete lists of upregulated and downregulated genes for each of the top ten pathways are provided in Table 1 and Table 2.

### 3.2. Methylation Status of lncRNA and mRNA

**Figure 3:**
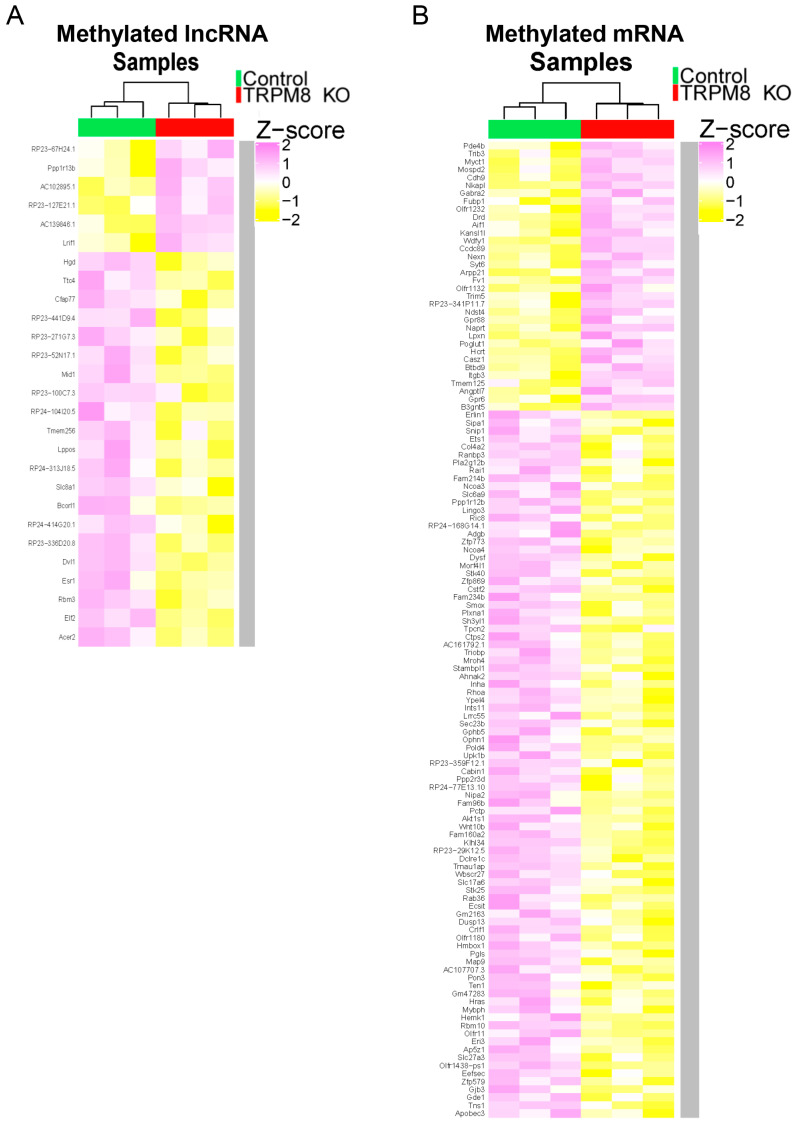
Methylation sequencing analysis: (**A**). Heatmap indicates the z-score of the genes corresponding to the methylated lncRNA in TRPM8 KO brains compared to the WT. (**B**). Heatmap indicates the z-score of the genes corresponding to the methylated mRNA in TRPM8 KO brains compared to the WT.

**Figure 4:**
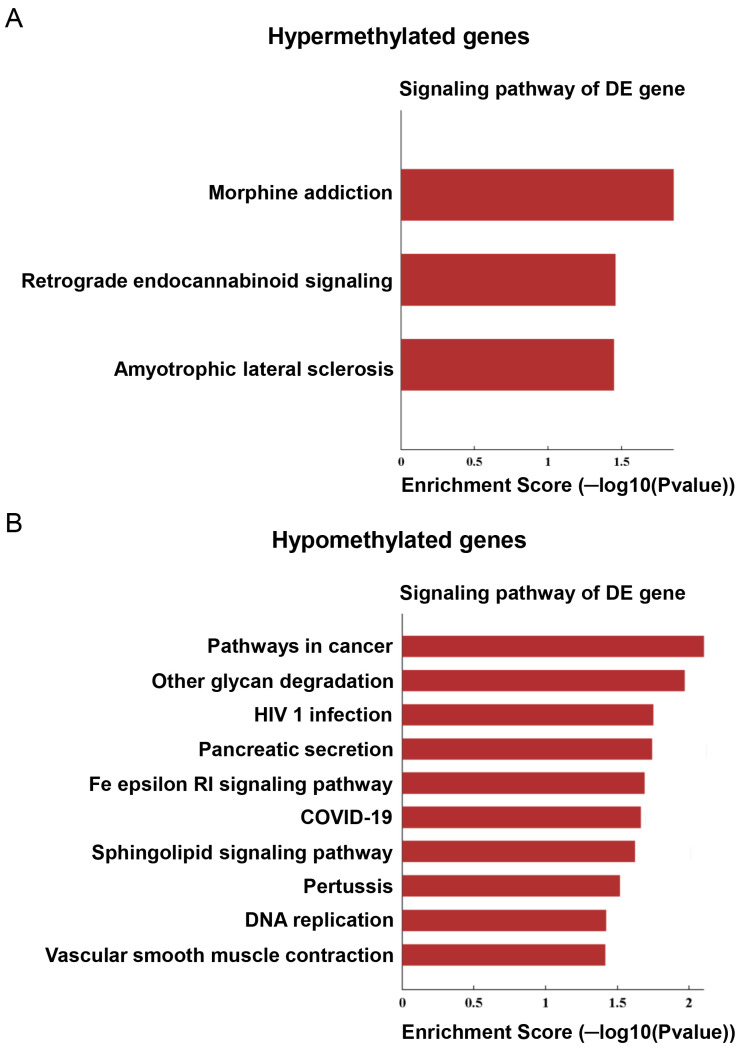
Pathway analysis of hypermethylation and hypomethylated mRNA: (**A**). Signaling pathway analysis showing enrichment score of genes in hypermethylated mRNA of TRPM8 KO mice brains compared to WT. (**B**). Signaling pathway analysis showing enrichment score of genes in hypomethylated mRNA of TRPM8 KO mice brains compared to WT.

The same mRNA and lncRNA samples were also subject to microarray analysis following immunoprecipitation by the anti-m6A antibody, aiming to delineate the differences in the methylation status between WT and TRPM8 KO mouse brains. The differentially methylated genes were identified and shown in the heatmaps generated by employing the ComplexHeatmap library (Figure 3A,B) (Supplementary Table S2). Interestingly, substantially more hypomethylated genes than hypermethylated genes were found in both lncRNAs and mRNAs from the TRPM8 KO brains. The signaling pathways associated with the identified genes, pooled between lncRNA and mRNA (Figure 4A,B), were mapped to the KEGG pathway database based on criteria established in prior research. The hypermethylated genes are mainly linked to three pathways: morphine addiction, retrograde endocannabinoid signaling, and amyotrophic lateral sclerosis (Figure 4A). By contrast, the hypomethylated genes primarily belong to ten distinct pathways, which are related to cancer, glycan degradation, HIV-1 infection, pancreatic secretion, Fc epsilon RI signaling, coronavirus, sphingolipid signaling, pertussis, DNA replication, and vascular smooth muscle contraction (Figure 4B). Detailed information concerning specific genes and their corresponding *p*-values is provided in Table 3 and Table 4.

**Figure 5:**
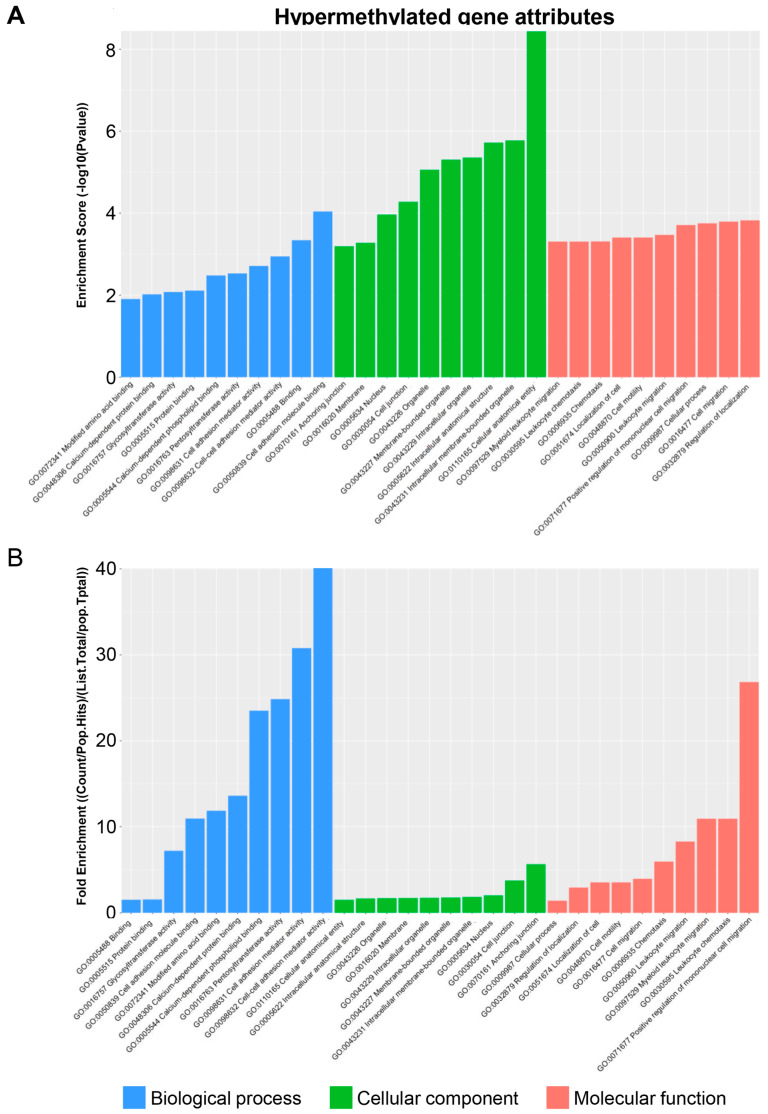
Attribute analysis of hypermethylated genes in TRPM8 KO male mice brain: (**A**). GO functional analysis of hypermethylated genes, showing enrichment scores for biological processes, cellular components, and molecular function. (**B**). GO functional analysis of hypermethylated genes, showing fold enrichment scores for biological processes, cellular components, and molecular function.

To deepen our understanding of the functional relevance of the identified hyper- and hypomethylated genes identified, we conducted a GO analysis. This analysis aims to unveil any unforeseen genes associated with processes pertinent to future research endeavors. Moreover, it is crucial to elucidate which biological processes and functions are altered in the TRPM8 KO model to prioritize the genes and pathways for subsequent investigation. The GO analysis of hypermethylated genes revealed the highest enrichment scores in cell adhesion molecule binding within the biological process domain and cellular–anatomical entity within the cellular component domain. With respect to fold enrichment, in line with TRPM8 being a calcium-permeable channel, calcium-related processes, along with cell adhesion and cell–cell adhesion mediator activities in the biological process domain, exhibited the most robust increases (Figure 5A,B). These changes suggest that TRPM8 deficiency potentially impairs synaptic plasticity or the formation of neural networks within the brain [40,41,42].

**Figure 6:**
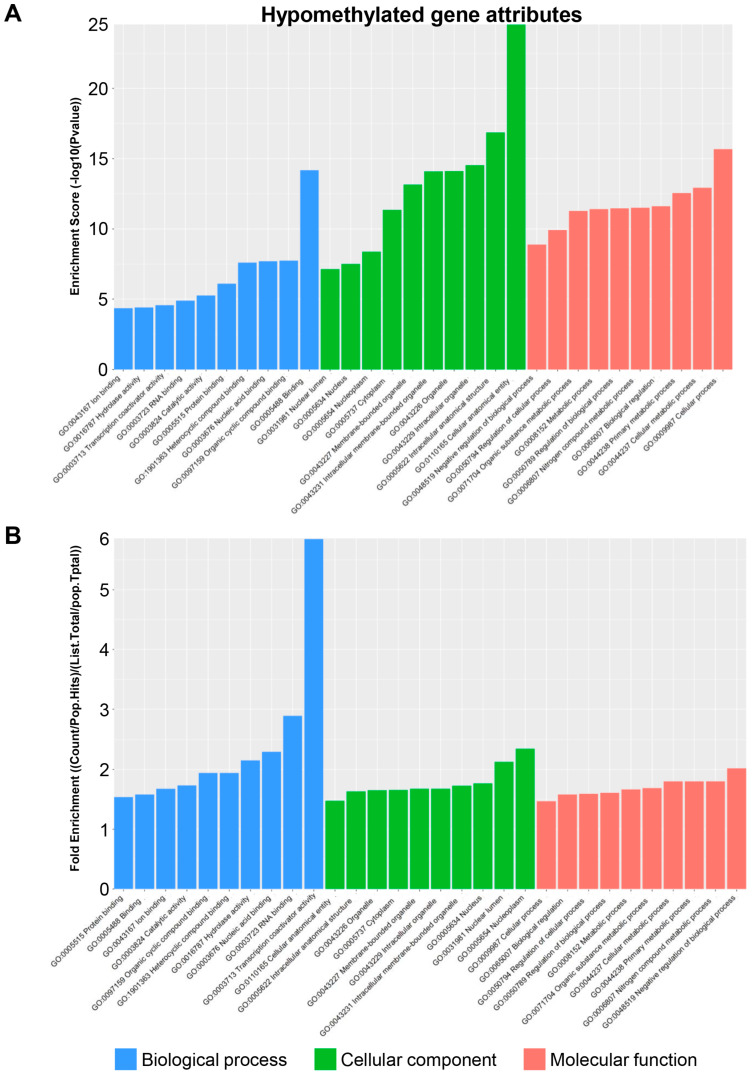
Attribute analysis of hypomethylated genes in TRPM8 KO male mice brain: (**A**). GO functional analysis of hypomethylated genes, showing enrichment scores for biological processes, cellular components, and molecular function. (**B**). GO functional analysis of hypomethylated genes, showing fold enrichment scores for biological processes, cellular components, and molecular function.

**Figure 7:**
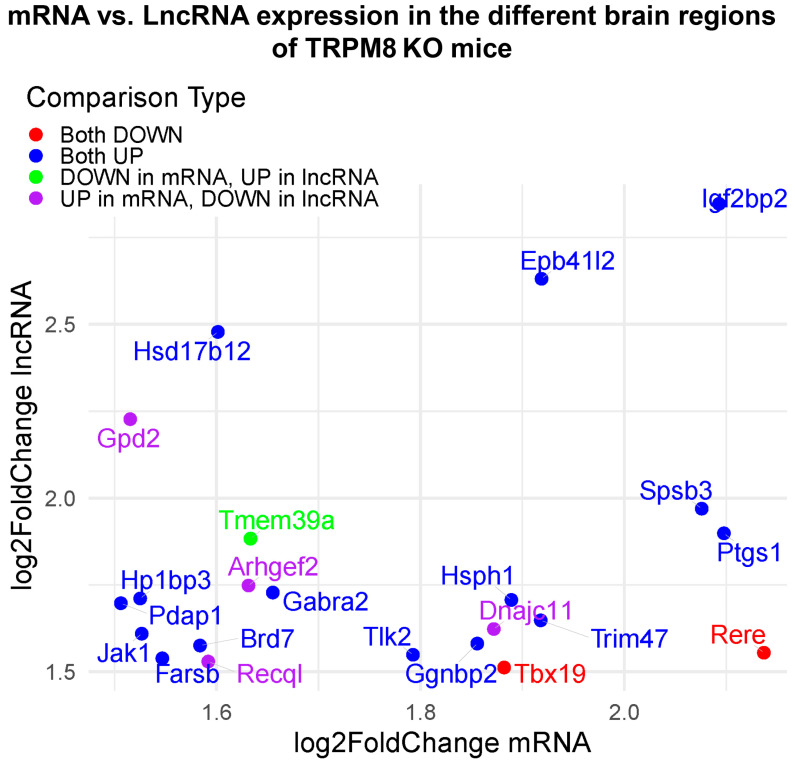
Scatter plot comparing mRNA and lncRNA expression in TRPM8 KO mouse brain regions. This scatter plot illustrates the log2 fold changes in gene expression of mRNA and lncRNA under specific experimental conditions. Each data point represents a gene, with the horizontal axis indicating the fold change in mRNA and the vertical axis representing the fold change in lncRNA. The color scheme represents different patterns of change (red points indicate genes that are downregulated in both mRNA and lncRNA; blue points signify genes that are upregulated in both; green points denote genes that are downregulated in mRNA but upregulated in lncRNA; purple points represent genes that are upregulated in mRNA but downregulated in lncRNA). Noteworthy genes are explicitly labeled on the plot, highlighting significant alterations in their expression levels.

The GO analysis of hypomethylated genes indicated a high enrichment score in binding within the biological process domain, the cellular–anatomical entity within the cellular component domain, and cellular processes within the molecular function domain (Figure 6A). Thus, in the absence of TRPM8, genes related to both binding activity and anatomical entities of the cell appear to be affected. An examination of the enrichment fold change of the hypomethylated genes revealed that the transcription coactivator activity within the biological process domain was the most affected gene set (Figure 6B). This suggests activation of a broad range of genes in the TRPM8-deficient brain, which may underly many of the neuronal activity changes in the mutant mice. Within the cellular component and molecular function domains, the highest increases were found in the nucleoplasm and negative regulation of the biological process, respectively, but the differences were marginal compared to other gene sets (Figure 6B). Furthermore, Figure 7 presents a comprehensive summary of gene classifications, identifying them as upregulated, downregulated, or exhibiting both characteristics within lncRNA or mRNA contexts. This detailed analysis intends to provide significant insights relevant to future experimental endeavors.

### 3.3. Distribution of AR-ER Pathway and Steroidogenesis Pathway Genes Between WT and TRPM8 Genes

To investigate the mechanisms underlying differential gene expression between WT and TRPM8 KO mouse brains, we selected specific Gene Ontology (GO) terms from the Amigo database. These terms were utilized to correlate the genes in the differential gene expression list. Then, we plotted the expression levels between WT and TRPM8 KO mice for lncRNAs and mRNAs separately.

**Figure 8:**
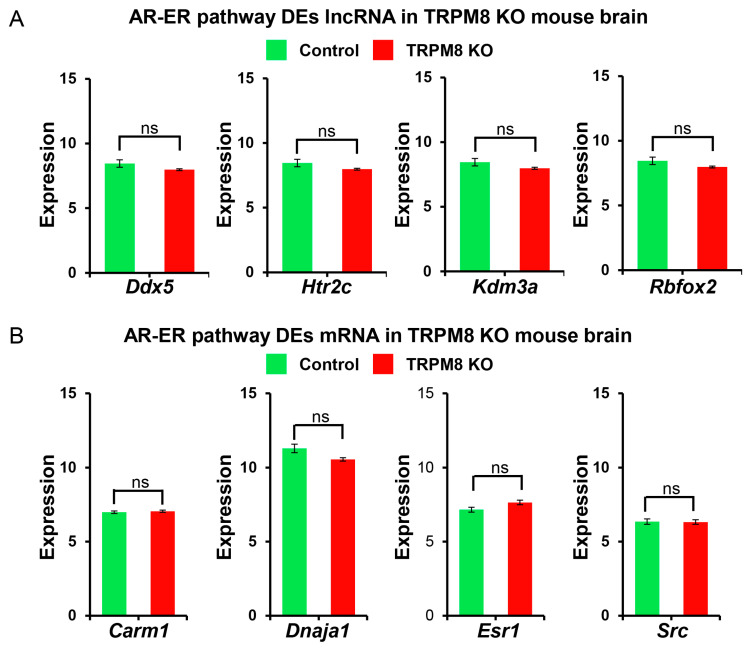
AR-ER pathway lncRNA and mRNA analysis in TRPM8 KO mice brains: (**A**). Comparison of differently expressed lncRNA genes between TRPM8 KO genes and wild-type control (Student’s *t*-test: ns, *p* > 0.05; *n* = 3). (**B**). Comparison of differently expressed mRNA genes between TRPM8 KO genes and wild-type control (Student’s *t*-test: ns, *p* > 0.05; *n* = 3).

**Figure 9:**
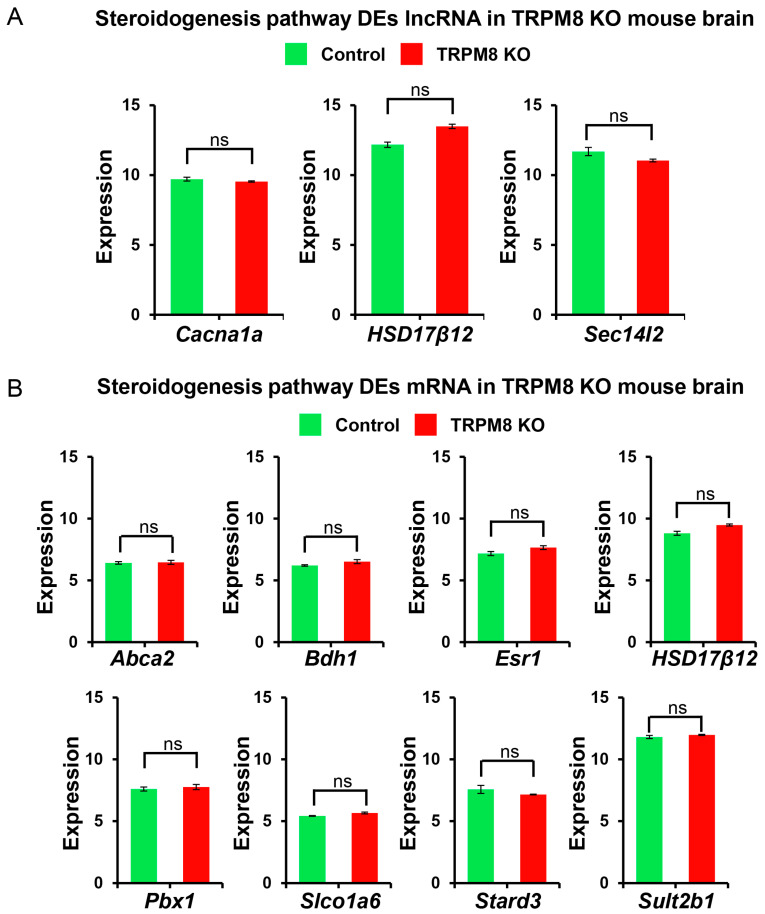
Steroidogenesis pathway lncRNA and mRNA analysis in TRPM8 KO mice brains: (**A**). Comparison of differently expressed lncRNA genes between TRPM8 KO genes and WT control (Student’s *t*-test: ns, *p* > 0.05; *n* = 3). (**B**). Comparison of differently expressed mRNA genes between TRPM8 KO genes and WT control (Student’s *t*-test: ns, *p* > 0.05; *n* = 3).

We first concentrated on the androgen receptor–estrogen receptor (AR-ER) signaling pathway because of the dysfunctions in sexual behavior found in the TRPM8 KO mice [10]. The analysis of the lncRNA data uncovered differential distributions of *Ddx5*, *Htr2c*, *Kdm3a*, and *Rbfox2* (Figure 8A), while that of the mRNA data indicated differential distributions of *Carm1*, *Dnaja1*, *Esr1*, and *Src* genes (Figure 8B). We then extended our evaluation to genes associated with the steroidogenesis pathway as changes in steroidogenesis were also found in the TRPM8 KO mice [10]. The lncRNA analysis unveiled *Cacna1a*, *HSD17β12*, and *Sec14l2* as the significantly altered genes (Figure 9A). In the mRNA analysis, however, differential distributions were found to include *Abca2*, *Bdh1*, *Esr1*, *HSD17β12*, *Pbx1*, *Slco1a6*, *Stard3*, and *Sult2b1* in the brains of WT and TRPM8 KO mice (Figure 9B).

### 3.4. Distribution of Sexual Reward Pathway and Cocaine Reward Pathway Genes Between WT and TRPM8

**Figure 10:**
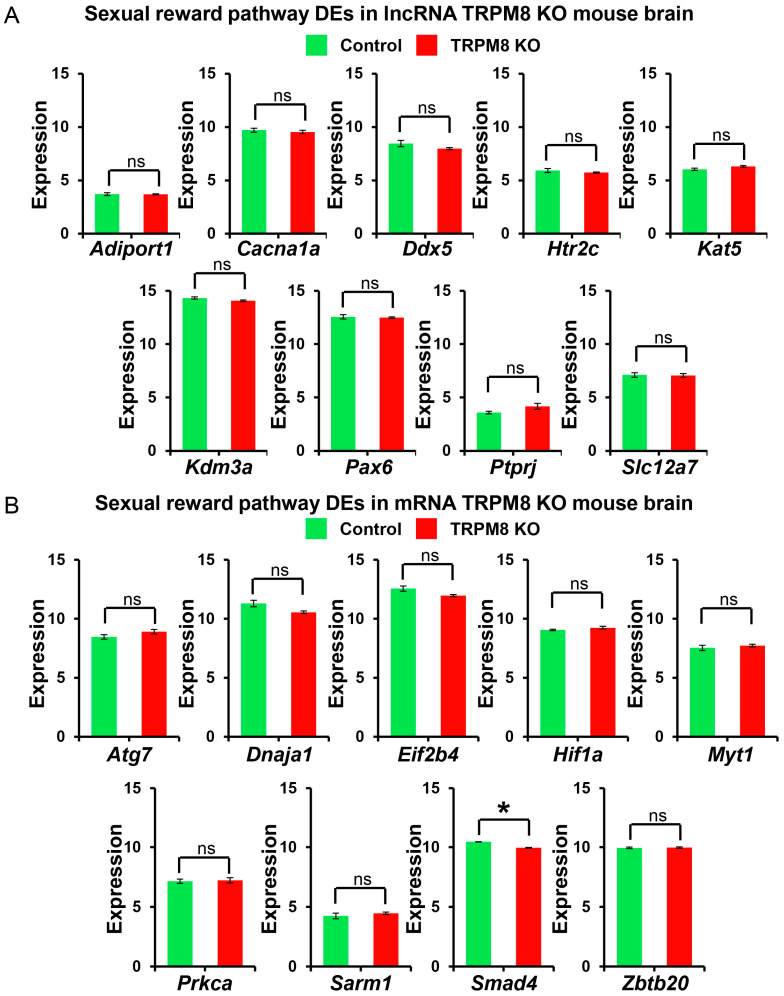
Sexually related pathway lncRNA and mRNA analysis in TRPM8 KO mice brains: (**A**). Comparison of differently expressed lncRNA genes between TRPM8 KO genes and WT control (Student’s *t*-test: ns, *p* > 0.05; *n* = 3). (**B**). Comparison of differently expressed mRNA genes between TRPM8 KO genes and WT control (Student’s *t*-test: ns, *p* > 0.05; *, *p* < 0.05; *n* = 3).

Next, we investigated the transcriptomic changes in the reward pathways, owing to modifications found in sexual satiety among TRPM8 KO mice [10]. Significant alterations within the sexual reward pathways were noticed in both lncRNA and mRNA of the TRPM8 KO samples. The analysis of the lncRNA data identified *Adiport1*, *Cacna1a*, *Ddx5*, *Htr2c*, *Kat5*, *Kdm3a*, *Pax6*, *Ptpri*, and *Slc2a7* as the primary DEGs (Figure 10A). The analysis of the mRNA data highlighted *Atg7*, *Dnaja1*, *Eif2b4*, *Hif1a*, *Myt1*, *Prka*, *Sarm1*, *Smad4*, and *Zbtb20* as significant genes of interest (Figure 10B).

**Figure 11:**
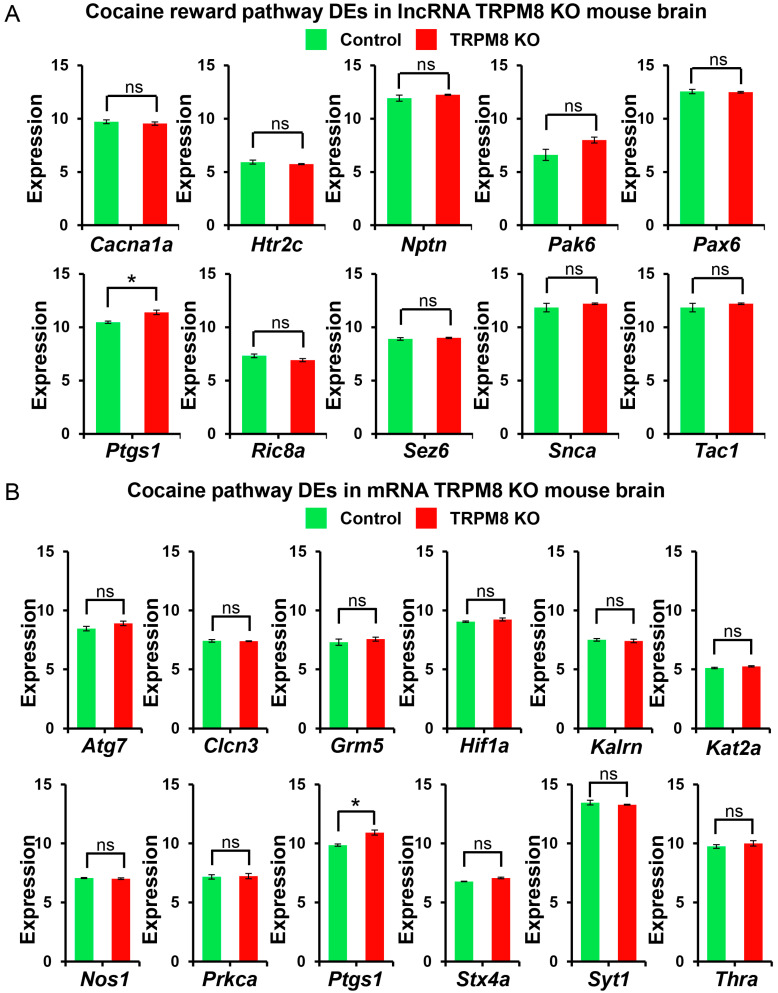
Cocaine reward pathway lncRNA and mRNA analysis in TRPM8 KO mice brains: (**A**). Comparison of differently expressed lncRNA genes between TRPM8 KO genes and WT control (Student’s *t*-test: ns, *p* > 0.05; *, *p* < 0.05; *n* = 3). (**B**). Comparison of differently expressed mRNA genes between TRPM8 KO genes and WT control (Student’s *t*-test: ns, *p* > 0.05; *, *p* < 0.05; *n* = 3).

To further explore the role of TRPM8 in reward pathways, we also investigated other reward systems. Within the lncRNA data, *Cacna1a*, *Htr2c*, *Nptn*, *Pak6*, *Pax6*, *Ptgs1*, *Ric8a*, *Snca*, and *Tac1* were significantly altered (Figure 11A). Notably, we also observed *Cacna1a*, *Htr2c*, and *Pax6* in the context of the sexual reward pathway. The Voltage-Gated Calcium Channel Subunit Alpha1 (*Cacna1a*) is of particular relevance due to its role in neurotransmitter release and its involvement in the processes of long-term potentiation and long-term depression [43]. These channels are widely expressed in neurons of many brain regions, including the ventral tegmental area (VTA), where reward processing occurs [44,45,46,47]. The 5-hydroxytryptamine receptor 2C (*Htr2c*) is expressed in the hypothalamus, hippocampus, cortex, and amygdala [48,49]. This receptor inhibits the release of both dopamine and serotonin in the VTA and amygdala, implicating its significance in both reward systems [50,51]. Lastly, the Paired Box Gene 6 (*Pax6*) is a transcription factor that plays a critical role during development, particularly in properly forming the central cortex [52,53]. The disruption of *Pax6* has also been linked to major depressive disorder [54].

The mRNA data yielded the following genes of interest within the cocaine reward pathway affected by the deletion of TRPM8: *Atg7*, *Clcn3*, *Grm5*, *Hif1a*, *Kalrn*, *Kat2a*, *Nos1*, *Prkca*, *Ptgs1*, *Stx4a*, *Syt1*, and *Thra* (Figure 11B). Consistent with our findings in the lncRNA data, several of the mRNA DEGs identified in the sexual reward pathway were found in the cocaine reward pathway as well. These genes included Autophagy Related 7 (*Atg7*), Hypoxia-Inducible Factor 1 Subunit Alpha (*Hif1a*), and Protein Kinase C Alpha (*Prkca*). In contrast to the prevalent relevance of the DEGs found in the lncRNA data, little is known about the roles of these genes in reward mechanisms, although both *Atg7* and *Prkca* have been associated with neurodegenerative diseases [55,56,57,58].

## 4. Discussion

RNA analysis serves as a robust approach for assessing gene expression, characterized by its extensive coverage and reliability. The genetic ablation of TRPM8 in mice leads to significant behavioral and developmental changes, as well as alterations in the expression of multiple genes. While prior studies have elucidated the functionalities affected by the deficiency of this channel [10,59,60], a comprehensive RNA analysis of the DEGs in the brain tissues of TRPM8-deficient mice remains to be conducted. This study aimed to fill this gap by examining the expression of lncRNA and mRNA in brain tissues between WT and TRPM8 KO mice, allowing for the compilation of a comprehensive list of DEGs. We identified a considerable number of upregulated and downregulated genes from the TRPM8 mutant brain tissues, along with the identification of numerous pathways influenced by this channel. Notably, we observed a spectrum of upregulated genes implicated in neuroactive ligand–receptor interactions and several downregulated genes associated with cancer signaling pathways. Additionally, our investigation into RNA methylation indicated a higher prevalence of mRNA methylation than lncRNA methylation in both WT and TRPM8 KO brain tissues, and TRPM8 deletion generally leads to more hypomethylation than hypermethylation. This post-transcriptional modification suggests enhanced susceptibility to epigenetic regulation at the RNA level in the absence of the TRPM8 channel.

KEGG analysis of the differentially methylated genes also revealed that the hypomethylated signaling pathways outnumber the hypermethylated pathways in the TRPM8 KO brain tissues. The hypermethylated genes are associated with specific signaling pathways such as morphine addiction, retrograde endocannabinoid signaling, and amyotrophic lateral sclerosis. In contrast, the hypomethylated genes fall into a wider array of pathways, encompassing cancer pathways, HIV-1 infection, COVID-19, and several others. This indicates an increase in genomic instability in the mutant mice. The DEGs within these pathways warrant further investigation to elucidate the mechanisms underlying the physiological and behavioral alterations found in TRPM8 mutants and to identify potential therapeutic targets.

The enrichment analysis of hypermethylated genes revealed that key cellular attributes, anatomical entity, and cell–cell adhesion mediator activity are significantly disrupted in the TRPM8 knockout mice. In parallel, the analysis of hypomethylated genes indicated their influence on binding attributes and transcription coactivator activity. The extensive array of pathways impacted by the lack of TRPM8 underscores its potential significance in these biological mechanisms; however, further research is warranted to elucidate the precise role of TRPM8 within the aforementioned pathways.

Because of the involvement of TRPM8 in sexual behavior and reward, we also specifically analyzed the DEGs in lncRNA and mRNA in four key pathways relevant to these functions: AR-ER, steroidogenesis, sexual reward, and cocaine reward pathways. We identified four common genes exhibiting differential expression across the AR-ER pathway, the sexual reward pathway, and the cocaine reward pathway: *Ddx5*, *Htr2c*, *Kdm3a*, and *Dnaja1*. Consistent with TRPM8 playing pivotal roles in sex responses, these genes are known to regulate the expression, trafficking, and/or activity of the sex hormone receptors, AR, and ER.

DEAD-Box Helicase 5 (*Ddx5*) is a member of the DEAD-box RNA helicase family, which plays a crucial role in brain function, behavior, and hormonal signaling, particularly in association with androgen and estrogen receptors [61]. Its involvement in various signaling pathways and regulatory processes holds significant implications for understanding reward-related behaviors, including sexual behavior and drug addiction. Moreover, Ddx5 has been implicated in the regulation of gene expression during neurodevelopment, interacting with RNA-binding proteins to modulate the splicing of mRNAs that are critical for neuronal function [62,63]. Additionally, Ddx5 functions as a transcriptional co-regulator for both androgen and estrogen receptors, thereby influencing their signaling pathways [64]. It has also been demonstrated that Ddx5 interacts with these receptors to facilitate their recruitment to the target gene promoters [61]. The 5-hydroxytryptamine receptor 2C (*Htr2c*), a serotonin receptor, is predominantly expressed in the brain and interacts with both AR and ER, influencing their signaling pathways [65,66]. Sex hormones can modulate this receptor, impacting the expression of genes regulated by AR and ER, which in turn affects sexual differentiation and reproductive behaviors. For example, estrogen enhances the expression and activity of Htr2c, potentially influencing behaviors related to sexual motivation and aggression [67].

Lysine demethylase 3A (*Kdm3a*), also referred to as JMJD1A, is a histone demethylase that plays a pivotal role in the central nervous system and hormonal signaling [68,69]. Through the mechanism of epigenetic modification, Kdm3a regulates gene expression, rendering it a significant factor in regulating various neurological functions, particularly in relation to androgen and estrogen receptors. Numerous studies have demonstrated that Kdm3a enhances the activity of these receptors by specifically demethylating H3K9, thus promoting the expression of AR target genes [70]. This is particularly relevant in the context of prostate cancer, where Kdm3a regulates the transcriptional program of AR, facilitating tumor growth and progression [70]. Additionally, Kdm3a has been found to influence estrogen signaling, particularly in its role in the effectiveness of ER signaling in breast cancer cells, suggesting a role in mediating the effects of both sex hormones on gene expression [71,72]. On the other hand, Dnaja1 serves as a co-chaperone for AR, facilitating its proper folding and function [73]. Dnaja1 has also been implicated in regulating ER-mediated transcriptional activation and is known to enhance the expression of estrogen-responsive genes [74,75].

Additionally, our analysis identified a single DEG common to both the AR-ER and steroidogenesis pathways: the estrogen receptor alpha (*Esr1*) gene. Esr1 is extensively recognized for its pivotal role in mediating estrogen signaling; however, it also engages in interactions with androgen receptors across various biological contexts. Notably, the presence of ESR1 within specific regions of the brain can modulate the effects of androgens, consequently influencing behaviors associated with male aggression and mating [76]. Neurons that are Esr1-positive and project from the medial preoptic area are recognized for their role in innervating dopaminergic neurons in the ventral tegmental area (VTA), thereby influencing the sexual activity of male mice [77].

The Voltage-Gated Calcium Channel Subunit Alpha1 A (*Cacna1a*) gene represents a notable commonality across the pathways involved in steroidogenesis, sexual reward, and cocaine reward. Cacna1a, categorized as a P/Q-type calcium channel, plays a crucial role in the dopaminergic pathways associated with reward processing. Current research indicates that variations in Cacna1a expression may significantly influence the brain’s response to rewarding stimuli, including sexual cues and psychoactive substances [78,79]. For example, mutations within this gene may result in alterations to dopaminergic signaling, which plays a critical role in the reinforcing impacts of drugs [80].

Intriguingly, the steroidogenesis pathway stands out as the only one with a DEG identified in both the lncRNA and the mRNA datasets: the hydroxysteroid 17-β dehydrogenase 12 (*HSD17β12*) gene. HSD17β12 is a member of the hydroxysteroid dehydrogenase family, which plays a critical role in steroid hormone metabolism, potentially impacting brain function, behavior, and hormone signaling. Its primary function involves converting 17-keto steroids to their corresponding 17-hydroxy forms, which is essential for sex hormone synthesis [81]. This enzymatic activity is crucial for maintaining hormonal balance, mood regulation, and reproductive behaviors. Studies have shown that changes in the expression of HSD17β12 can influence the brain’s response to reward cues [82].

Notably, some of the DEGs identified here are shared among multiple pathways. This is not surprising because many genes play functions in multiple pathways, but the identification of DEGs nevertheless presents additional avenues for further investigation. It is essential to emphasize that our findings align with the prior characterization of TRPM8 mutant mice [9,10], underscoring the necessity for further behavioral and functional studies to elucidate the relationships between TRPM8 and the genes identified in this study. This endeavor holds the promise of unveiling novel targets for therapeutic development.

**Figure 12:**
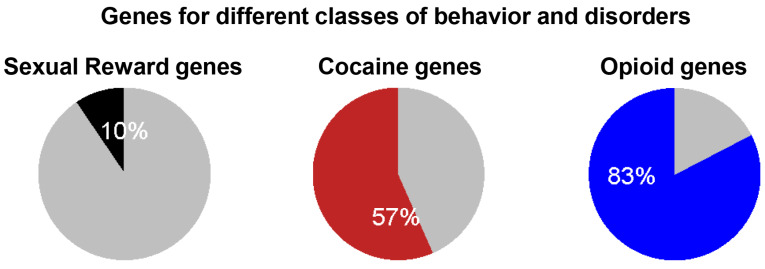
Percentage of overlapping genes for the differential class of disorders in TRPM8 KO mice brains. The genes associated with different behaviors and disorders were compiled from various studies. Gene lists were extracted from papers or Supplementary Files, and for studies that did not provide gene lists, the GEO2R function was used to identify differentially expressed genes. The resulting list of overlapping genes between each behavior or disorder and TRPM8 KO was filtered based on a *p*-value of 0.05 and a fold change of |1.5|, then further refined by removing redundant genes. These filtered gene lists were then compared to the differential gene list of mouse brain data to identify overlapping genes, and the results were used to create a distribution pie chart. Colored areas represent the percentage of overlapping genes for each disorder.

The diagrams in Figure 12 depict the fractions of the identified DEGs within the total complied list of genes known to be involved in the indicated biological function or disorder, such as the response to sex, cocaine, or opioids. By focusing on brain regions involved in reward for changes in lncRNA and mRNA expression, as well as their methylation in TRPM8 KO mice, our findings illuminate the intricate nature of gene regulation in the absence of the TRPM8 channel. Subsequent research should aim to elucidate the interactions between the identified genes and TRPM8 to better comprehend their roles in behavioral regulation.

## Figures and Tables

**Table 1:** List of upregulated genes and corresponding pathways.

Pathway ID	Definition	Fisher-*p*-Value	Genes
mmu04080	Neuroactive ligand-receptor interaction	0.000345744	ADRA2C//ADRB1//AGT//APLN//CNR1//DRD2//DRD5//EDN1//GABRA2//GABRA6//GNRH1//GRIK1//GRP//HCRT//HTR2C//HTR4//NPY2R//NPY5R//NR3C1//S1PR1//S1PR5//TAC1//TACR3
mmu00062	Fatty acid elongation	0.00113356	ACOT7//ECHS1//ELOVL1//ELOVL2//HSD17B12
mmu00920	Sulfur metabolism	0.003031833	SELENBP1//SELENBP2//SUOX
mmu03440	Homologous recombination	0.005446263	ABRAXAS1//BARD1//BLM//NBN//RAD52
mmu05134	Legionellosis	0.006426393	CD14//CYCS//ITGAM//NAIP1//SEC22B//TLR2
mmu00970	Aminoacyl-tRNA biosynthesis	0.01015479	FARS2//FARSB//GATC//HARS//SEPSECS//TARS
mmu01040	Biosynthesis of unsaturated fatty acids	0.01440289	ACOT7//ELOVL1//ELOVL2//HSD17B12
mmu00532	Glycosaminoglycan biosynthesis—chondroitin sulfate/dermatan sulfate	0.01739734	CHST7//CSGALNACT1//XYLT2
mmu05017	Spinocerebellar ataxia	0.01743527	ATG2A//ATP2A2//CYCS//KAT5//MAP3K5//PLCB4//PSMA3//PSMD2//WIPI2
mmu04924	Renin secretion	0.01830077	ADRB1//AGT//EDN1//KCNJ2//ORAI1//PLCB4
mmu05133	Pertussis	0.0205839	C1QA//C1QB//CD14//IRF8//ITGAM//TIRAP
mmu04145	Phagosome	0.02790083	CD14//H2-T24//ITGAM//NCF1//RAB5B//SEC22B//STX12//TAP2//THBS4//TLR2
mmu00534	Glycosaminoglycan biosynthesis—heparan sulfate/heparin	0.02846353	EXTL2//NDST4//XYLT2
mmu01212	Fatty acid metabolism	0.02935378	ACSF3//ECHS1//ELOVL1//ELOVL2//HSD17B12
mmu05152	Tuberculosis	0.02982701	CAMP//CD14//CYCS//HSPA9//IL10RB//ITGAM//JAK1//RAB5B//TIRAP//TLR2

**Table 2:** List of downregulated genes and corresponding pathways.

Pathway ID	Definition	Fisher-*p*-Value	Genes
mmu01522	Endocrine resistance	0.000886873	BCL2//DLL3//HRAS//MMP2//SHC2//SHC4
mmu04917	Prolactin signaling pathway	0.001962605	GSK3B//HRAS//SHC2//SHC4//TH
mmu01521	EGFR tyrosine kinase inhibitor resistance	0.00261813	BCL2//GSK3B//HRAS//SHC2//SHC4
mmu04722	Neurotrophin signaling pathway	0.003406531	BCL2//GSK3B//HRAS//NTRK2//SHC2//SHC4
mmu04012	ErbB signaling pathway	0.003419709	GSK3B//HRAS//PAK6//SHC2//SHC4
mmu05224	Breast cancer	0.008711338	BRCA1//DLL3//GSK3B//HRAS//SHC2//SHC4
mmu05226	Gastric cancer	0.009573469	BCL2//GSK3B//HRAS//SHC2//SHC4//TGFB1
mmu05220	Chronic myeloid leukemia	0.0133533	HRAS//SHC2//SHC4//TGFB1
mmu04672	Intestinal immune network for IgA production	0.01496991	AICDA//H2-EB1//TGFB1
mmu04926	Relaxin signaling pathway	0.019923	HRAS//MMP2//SHC2//SHC4//TGFB1
mmu00670	One carbon pool by folate	0.02169089	MTHFD1//MTHFR
mmu05210	Colorectal cancer	0.02175375	BCL2//GSK3B//HRAS//TGFB1
mmu04915	Estrogen signaling pathway	0.02305813	BCL2//HRAS//MMP2//SHC2//SHC4
mmu04910	Insulin signaling pathway	0.02649742	GSK3B//HRAS//PPP1R3B//SHC2//SHC4
mmu04933	AGE-RAGE signaling pathway in diabetic complications	0.03384903	BCL2//HRAS//MMP2//TGFB1
mmu04510	Focal adhesion	0.03487131	BCL2//GSK3B//HRAS//PAK6//SHC2//SHC4
mmu04730	Long-term depression	0.03584255	CACNA1A//CRHR1//HRAS
mmu03015	mRNA surveillance pathway	0.03599661	DDX19B//MAGOHB//MSI1//PABPC6
mmu04660	T cell receptor signaling pathway	0.03599661	GSK3B//HRAS//PAK6//PDCD1
mmu05310	Asthma	0.03631316	H2-EB1//IL9
mmu05321	Inflammatory bowel disease	0.03893883	H2-EB1//MAF//TGFB1
mmu05145	Toxoplasmosis	0.04412341	BCL2//H2-EB1//IRGM2//TGFB1
mmu04940	Type I diabetes mellitus	0.04552602	GM8909//H2-EB1//ICA1
mmu05211	Renal cell carcinoma	0.04901397	HRAS//PAK6//TGFB1

**Table 3:** List of hypermethylated genes and corresponding pathways.

Pathway ID	Definition	Fisher-*p* Value	Genes
mmu05032	Morphine addiction	0.01414558	GABRA2//PDE4B
mmu04723	Retrograde endocannabinoid signaling	0.03511991	GABRA2//NDUFB10
mmu05014	Amyotrophic lateral sclerosis	0.03580035	ANXA7//BECN1//NDUFB10

**Table 4:** List of hypomethylated genes and corresponding pathways.

Pathway ID	Definition	Fisher-*p* Value	Genes
mmu05200	Pathways in cancer	0.007956772	AGTR1A//CASP8//COL4A2//ETS1//HES5//HRAS//NCOA3//NCOA4//RHOA//TXNRD3//WNT10B
mmu00511	Other glycan degradation	0.01079583	MAN2B2//MANBA
mmu05170	HIV 1 infection	0.01790393	AP1G2//APOBEC3//CASP8//HRAS//IRAK1//IRF3
mmu04972	Pancreatic secretion	0.0182107	PLA2G12B//RHOA//SLC4A2//TPCN2
mmu04664	Fc epsilon RI signaling pathway	0.02048668	HRAS//INPP5D//MS4A2
mmu05171	Coronavirus disease	0.02187431	AGTR1A//IRAK1//IRF3//RPS15//RPS27//UBA52
mmu04071	Sphingolipid signaling pathway	0.02397238	HRAS//MS4A2//PPP2R3D//RHOA
mmu05133	Pertussis	0.03057378	IRAK1//IRF3//RHOA
mmu03030	DNA replication	0.03812052	POLD4//RNASEH2A
mmu04270	Vascular smooth muscle contraction	0.03850764	AGTR1A//PLA2G12B//PPP1R12B//RHOA
mmu05165	Human papillomavirus infection	0.03861895	CASP8//COL4A2//HES5//HRAS//IRF3//PPP2R3D//WNT10B
mmu03410	Base excision repair	0.04013575	APEX1//POLD4
mmu04614	Renin-angiotensin system	0.04013575	AGTR1A//KLK1B1
mmu05340	Primary immunodeficiency	0.04013575	AIRE//DCLRE1C
mmu05224	Breast cancer	0.04104249	HES5//HRAS//NCOA3//WNT10B
mmu05417	Lipid and atherosclerosis	0.04218974	CASP8//HRAS//IRAK1//IRF3//RHOA
mmu05216	Thyroid cancer	0.04219062	HRAS//NCOA4
mmu04072	Phospholipase D signaling pathway	0.04278418	AGTR1A//HRAS//MS4A2//RHOA

## Data Availability

The data will be publicly made available upon publication of this manuscript. The large data sequencing files will be shared upon valid request.

## Supplementary Materials

The following supporting information can be downloaded at: https://www.mdpi.com/article/10.3390/biomedicines13010075/s1, Table S1: Table of lncRNAs; Table S2: Table of mRNAs.
