# Supplementary material for: Transcriptomic Profile Analysis of Brain Tissue in the Absence of Functional TRPM8 Calcium Channel
Source: Biomedicines. 2024 Dec 31;13(1):75. doi: 10.3390/biomedicines13010075 (PMC11760472; doi:10.3390/biomedicines13010075)
Supplement: Supplementary file 1 [file biomedicines-13-00075-s001.zip › Supplementary Data.pdf]

Supplementary Data:

Supplementary Table S1. Excel file with the list of upregulated and downregulated lncRNAs and mRNAs.

Supplementary Table S2. Excel file with the list of methylated upregulated and downregulated lncRNAs and mRNAs.
